# Biomechanical Analysis of a Novel Prosthesis Based on the Physiological Curvature of Endplate for Cervical Disc Replacement

**DOI:** 10.1371/journal.pone.0158234

**Published:** 2016-06-29

**Authors:** Cheng-Cheng Yu, Ding-Jun Hao, Da-Geng Huang, Li-Xiong Qian, Hang Feng, Hou-Kun Li, Song-Chuan Zhao

**Affiliations:** Department of Spine Surgery, Honghui Hospital, Xi’an Jiaotong University Health Science Center, Xi’an, Shaanxi, China; Mayo Clinic Minnesota, UNITED STATES

## Abstract

**Study Design:**

Biomechanical analysis of a novel prosthesis based on the physiological curvature of endplate was performed.

**Objective:**

To compare the biomechanical differences between a novel prosthesis based on the physiological curvature of the endplate and the Prestige LP prosthesis after cervical disc replacement (CDR).

**Summary of Background Data:**

Artificial disc prostheses have been widely used to preserve the physiological function of treated and adjacent motion segments in CDR, while most of those present a flat surface instead of an arcuate surface which approximately similar to anatomic structures *in vivo*. We first reported a well-designed artificial disc prosthesis based on the physiological curvature of the endplate.

**Methods:**

Three motion segments of 24 ovine cervical spines (C2-5) were evaluated in a robotic spine system with axial compressive loads of 50N. Testing conditions were as follows: 1) intact, 2) C3–4 CDR with artificial disc prosthesis based on the physiological curvature of the endplate, and 3) C3–4 CDR with the Prestige LP prosthesis. The range of motion (ROM) and the pressures on the inferior surface of the two prostheses were recorded and analyzed.

**Results:**

As compared to the intact state, the ROM of all three segments had no significant difference in the replacement group. Additionally, there was no significant difference in ROM between the two prostheses. The mean pressure on the novel prosthesis was significantly less than the Prestige LP prosthesis.

**Conclusion:**

ROM in 3 groups (intact group, CDR group with novel prosthesis and CDR group with Prestige LP) showed no significant difference. The mean pressure on the inferior surface of the novel prosthesis was significantly lower than the Prestige LP prosthesis. Therefore, the novel artificial disc prosthesis is feasible and effective, and can reduce the implant-bone interface pressure on the endplate, which may be one possible reason of prosthesis subsidence.

## Introduction

As an alternative method to the standard anterior cervical discectomy and fusion (ACDF), cervical disc replacement (CDR) preserves segmental range of motion (ROM) and can avoid or mitigate degeneration of the adjacent segment [1–3]. Although CDR has greater benefits than fusion, it is associated with a new spectrum of complications [4, 5], including vertebral body fracture, dislocation, migration, bearing surface wear, and subsidence. Of these, subsidence is considered as one of the most common problems, with an incidence of approximately 3–10% [6–8]. Subsidence is the erosion of prostheses into the adjacent vertebral body. As compared to interbody fusion devices, which can be removed after fusion, the CDR prosthesis has to bear and transmit the axial load to the adjacent endplate for the duration of a patient’s life, which can lead to device subsidence [7, 9, 10]. The subsidence tendency is associated with high implant-bone interface pressure [11]. Additionally, reduced bone mineral density, poor endplate preparation, and uneven load distribution associated with the design of the prosthesis can accelerate subsidence [6, 8]. To avoid the occurrence of subsidence, careful preparation of the endplate and scientific design of the prosthesis are required. Surgeons should be cautious not to completely remove the endplate while polishing the vertebral body [8, 12]. Additionally, the footprint of a prosthesis should be large enough to dissipate rather than centralize the axial load [11].

**Fig 1:**
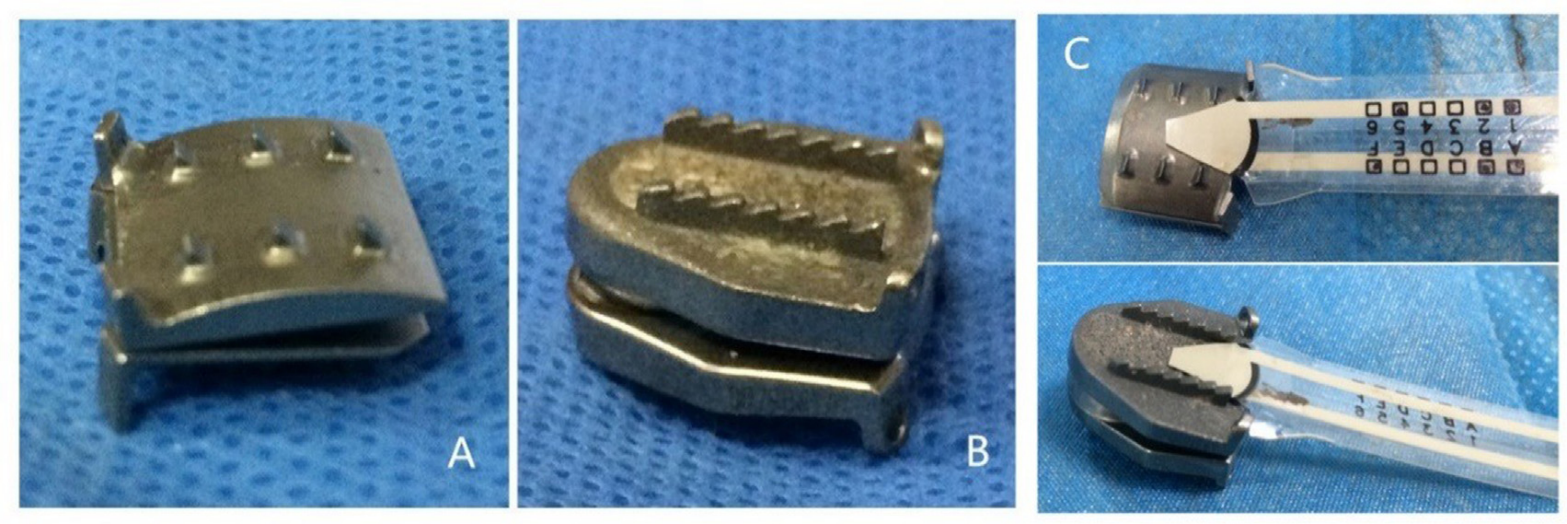
The prostheses. A. The novel cervical artificial disc prosthesis based on the physiological curvature of the endplate. B. Prestige LP prosthesis. C. Pressure measuring sensors on the inferior surface of the two prostheses.

The load on the prosthesis will be less if its surface is arcuate, like the curvature of the endplate, due to more contact area with the endplate and less polishing needed. However, most of artificial disc prostheses currently used present a flat surface instead of an arcuate surface. Therefore, we designed a novel artificial disc prosthesis based on the physiological curvature of the endplate (Fig 1A), in order to decrease subsidence and provide a better simulation of the real disc.

The Prestige LP (Medtronic Sofamor Danek Inc., USA) prosthesis (Fig 1B) has good clinical outcomes, preserves the overall cervical alignment and ROM of the treated and adjacent levels, and has been widely recognized worldwide [13]. The surface of the Prestige LP prosthesis is flat, similar to most prostheses currently used.

Human spinal specimens are often used for *in vitro* test. However, factors of human cadaver specimens, such as age, gender, height, bone quality and the grade of degeneration, confound the effects of the test. Additionally, human cadaver specimens are difficult to obtain. In contrast, spinal specimens from animals are more homogeneous and easier to obtain. The ovine cervical spine was recommended as an accepted model for human cervical spine research [14–16].

In this study, ovine cervical spines were utilized to quantify changes in kinematics. ROM and pressures on the inferior surface of two prostheses under the same conditions were compared. We tested the physiological motion function of the intact specimen and after the two prostheses were planted into the segments. We also tested whether the novel artificial cervical disc prosthesis based on the physiological curvature of the endplate can reduce the pressure on the inferior surface.

## Materials and Methods

### Specimen preparation

Twenty-four fresh frozen cadaveric cervical spines from two-year-old sheep were utilized in this study. The ovine spines were purchased from Shanghai Branch Center, Institute of Laboratory Animal Science, Chinese Academy of Medical Science. Before the biomechanical analysis, all specimens were evaluated for bone mineral density (BMD) using dual-energy x-ray absorptiometry scanning to ensure that none had pathologically low BMD (t-score > −2.5)[17]. After preparing for the biomechanical test, the specimens were frozen at -30 C, then thawed at room temperature for 24 hours before analysis. Care was taken to preserve the ligamentous attachments of each specimen, only muscular and fatty tissues were removed. C2-C5 of the ovine cervical spines were tested in a poly segmental setup. For stabilization, the proximal (C2) and distal (C5) ends of the specimen were embedded in cold curing resin adhesive (HEI-CAST 8012, Heisen Yoko Co.Ltd., Japan). The C2 was attached on the upper fixture and the T1 was mounted to the lower testing platform. Motion capture markers of the optical tracking system were inserted into the vertebral bodies of C2-C4.

### Three-dimensional motion testing

The proximal and distal ends of the specimen were mounted on a six axis spinal robot (Shanghai Sanyou Medical Co.Ltd., China). The moment arm attached to the proximal end of the specimen could apply axial load and the pure moment, whereas the distal end of the specimen remained fixed to the socket of the robot. The robot was programmed to apply three continuous loading-unloading cycles of applied moment along each primary axis of motion to simulate flexion-extension (FE), lateral bending (LB), and axial rotation (AR). An axial preload of 50N was given on the C2 to simulate the quality of head. All specimens were subjected to three cycles of FE, LB, and AR under the nondestructive pure moment of ± 2.0 Nm, and the data of the third cycle were used for analysis [18]. The ROM of the poly segment C2-5 was measured by the optical tracking system. During the biomechanical tests, all specimens were moistened with normal saline to prevent desiccation.

### Segmental ROM

**Fig 2:**
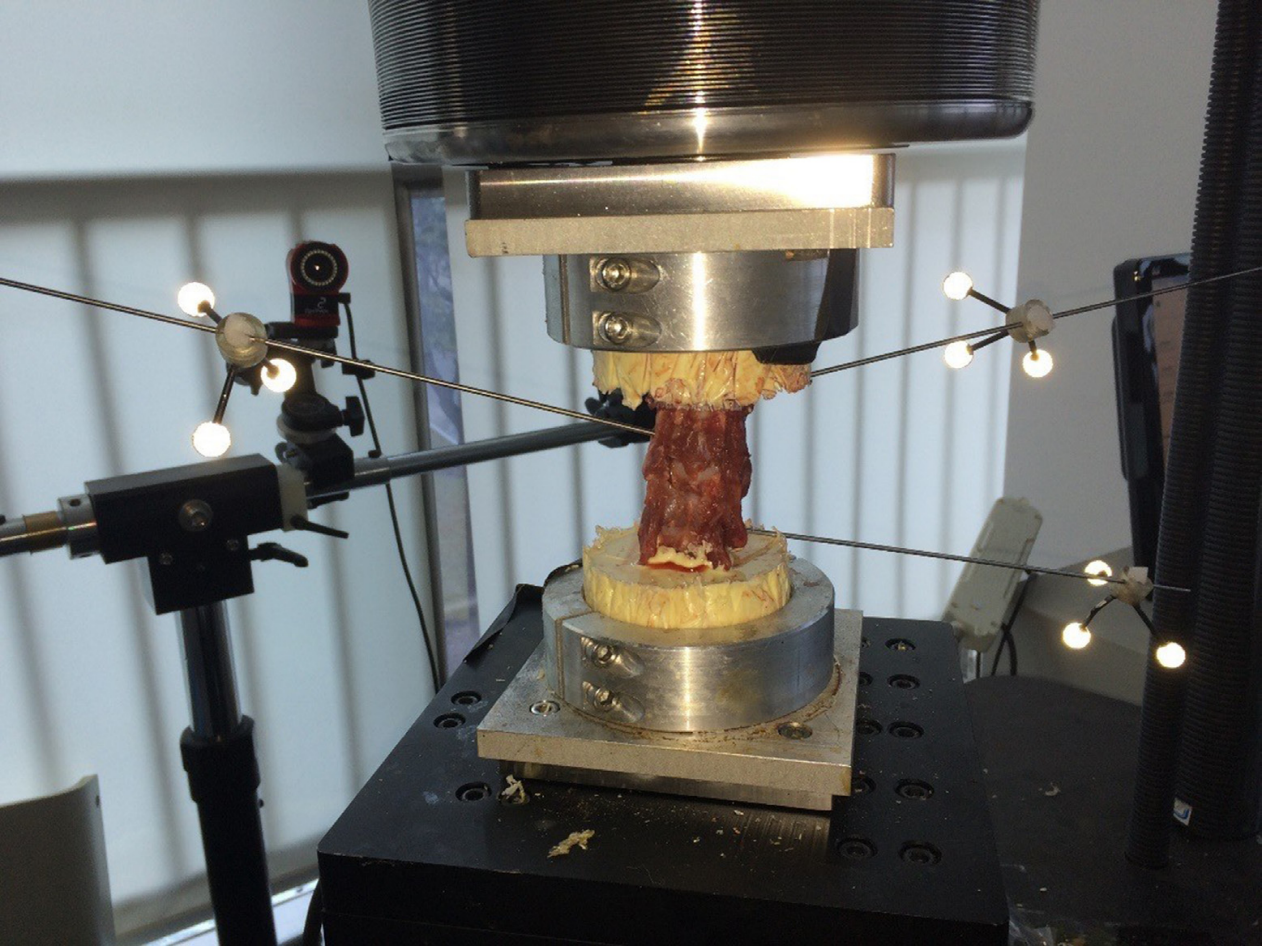
An intact spine specimen. Each rigid rod connected motion capture marker to the vertebral bodies of C2 (left side), C3 (right side) and C4 (left side) for the optical tracking system.

To evaluate the ROM in the C2/3, C3/4 and C4/5 segments, an optical tracking system (OptiTrack, NaturalPoint Inc., USA) was used. A rigid rod connected to the motion capture marker was inserted into the vertebral body of C2, C3 and C4 (Fig 2). Every motion capture marker consisted of three non-collinear optical balls, which could be detected by the optical tracking system. A marker was placed on the socket to attach C5 due to its immovability. The optical tracking system could directly measure the value of the angle.

### Pressure

Pressure measuring sensors (Flexiforce Pressure Sensor, Tekscan, Inc., USA) were placed on the inferior surface of the prostheses (Fig 1C). These sensors, which can tailor and calibrate, had the maximum pressure of 445N. The pressure data were continuously recorded on the computer.

### Reconstructive conditions

**Fig 3:**
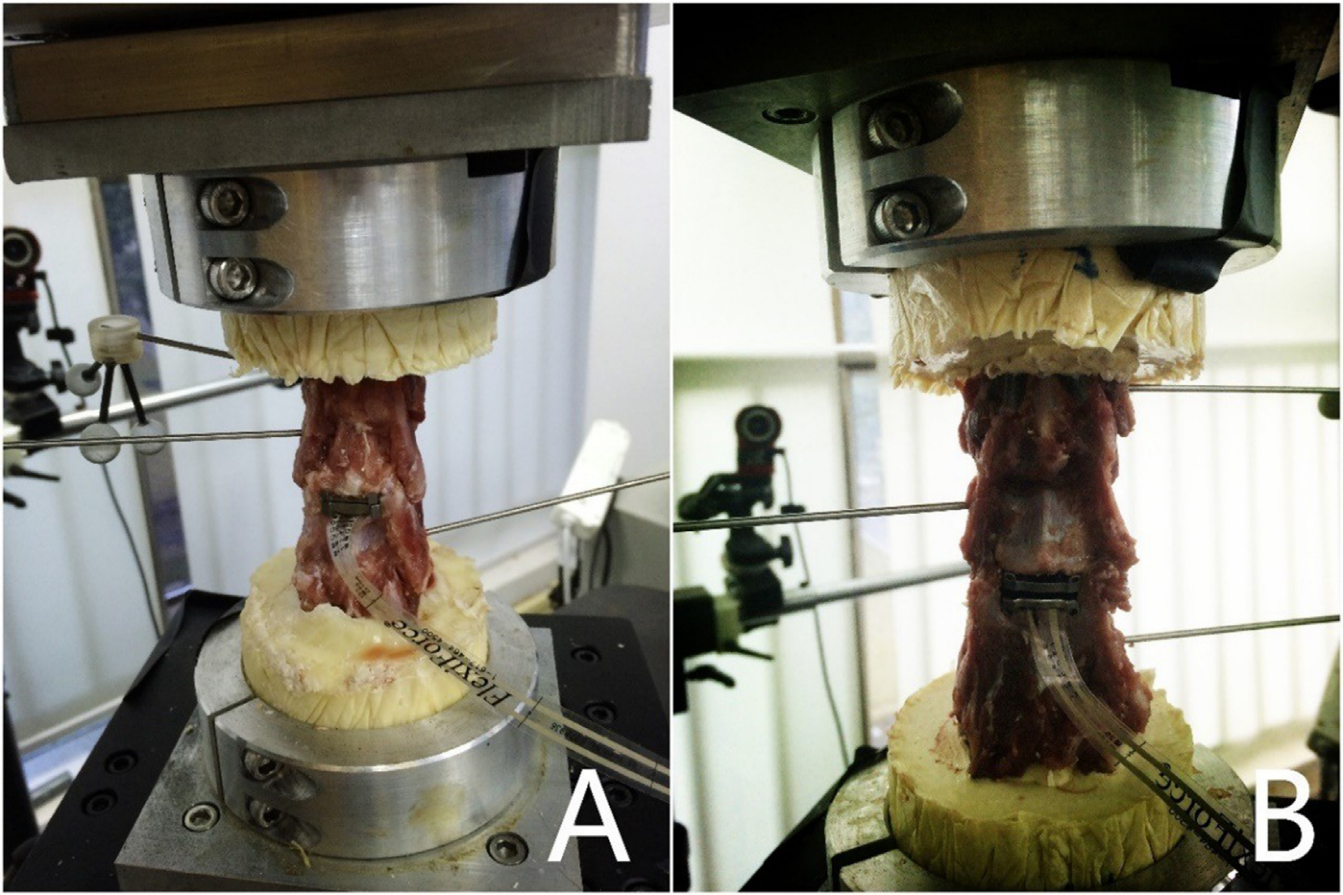
The specimens in the different conditions. A. C3/4 CDR with the novel cervical artificial disc prosthesis based on the physiological curvature of the endplate; B. C3/4 CDR with the Prestige LP prosthesis.

Twenty-four cervical spines were divided into three groups (group 1, 2 and 3), with eight specimens per group. A complete discectomy of C3/4 was performed in all spines of groups 2 and 3. The discectomy was performed by first removing the anterior longitudinal ligament. Next, a knife was placed into the C3/4 disc space to incise the annulus. Resection of the intervertebral disc was kept medial to the uncinate processes, which were preserved. The vertebral endplates were exposed by resection of the intervertebral disc, cleaned, but the bony surfaces preserved. The posterior longitudinal ligament was preserved because of its pivotal role in postsurgical stability [19, 20]. Thereafter, the endplates of the spines of the two groups were prepared using a high-speed burr to ensure adequate implant positioning. Trial sizes were used to estimate the size of the prosthesis that best fits each specimen. The novel prosthesis based on the physiological curvature of the endplate was inserted at the C3/4 level in group 2 (Fig 3A). While in group 3, the Prestige LP prosthesis was inserted at the same level (Fig 3B). The specimens of the three groups were analyzed.

### Radiographic control

**Fig 4:**
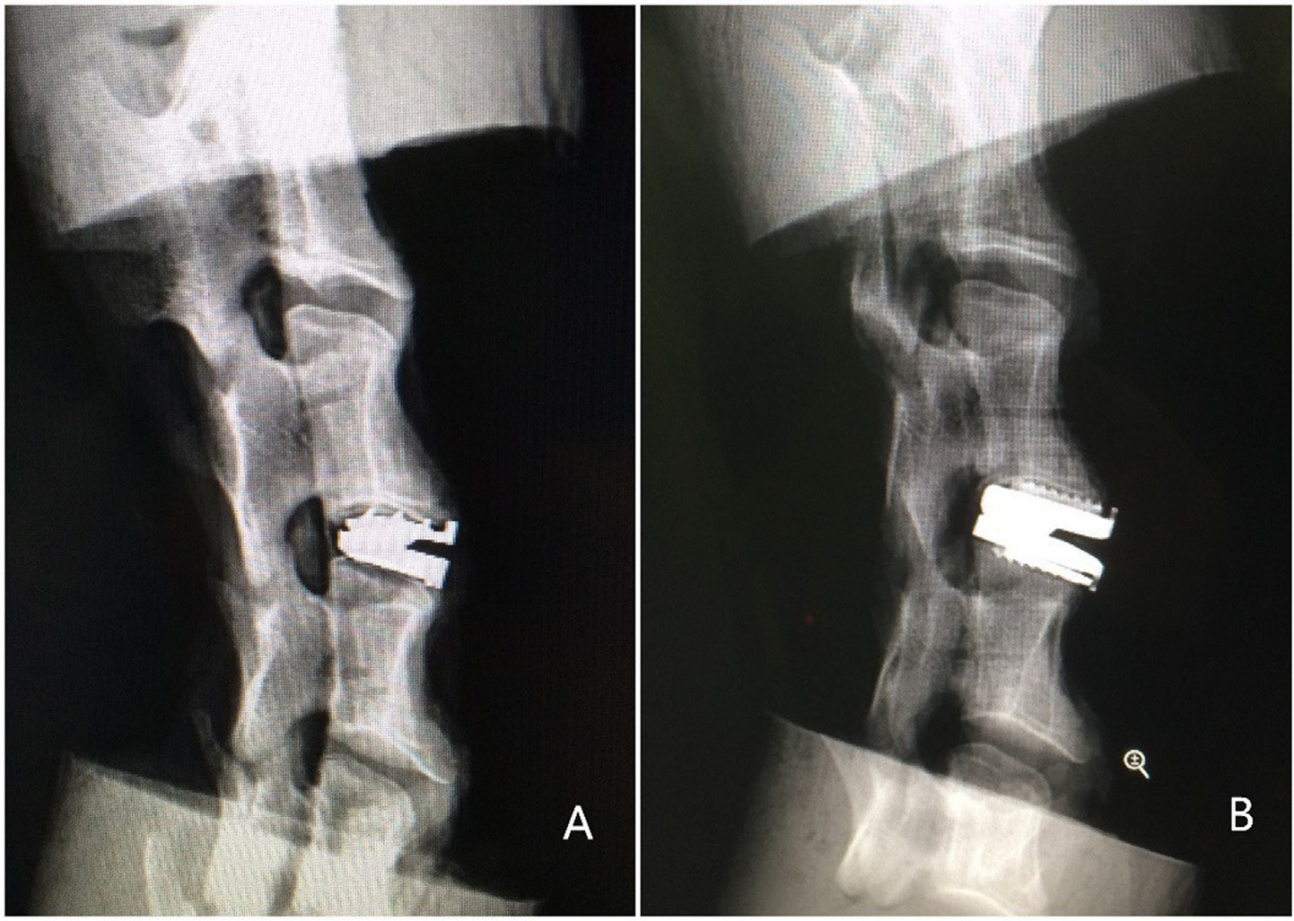
Radiographs of two different specimens. The correct position of the novel prosthesis (A) and the Prestige LP prosthesis (B).

Radiographs were taken to ensure correct positioning of the implants in the intervertebral space (Fig 4).

### Data and statistical analysis

For statistical analysis, the data of the third loading cycle for the six spinal motions were used. The total ROM and the ROM at C2/3, C3/4 and C4/5 segments were quantified at maximum load. The ROM values were compared among the three groups. The maximum pressures on the two prostheses’ inferior surfaces were compared. One-way ANOVA was used to determine the statistical differences in the three groups. Differences between the pressures were calculated using an unpaired Student’s t-test. A significance level of p < 0.05 was applied. Statistical analyses were performed using SPSS for Windows (version 19.0; SPSS, Inc., USA).

## Results

After biomechanical testing, all specimens in groups 2 and 3 were dissected and visually evaluated for damage. No fractures or hardware failure was noted.

### ROM

**Fig 5:**
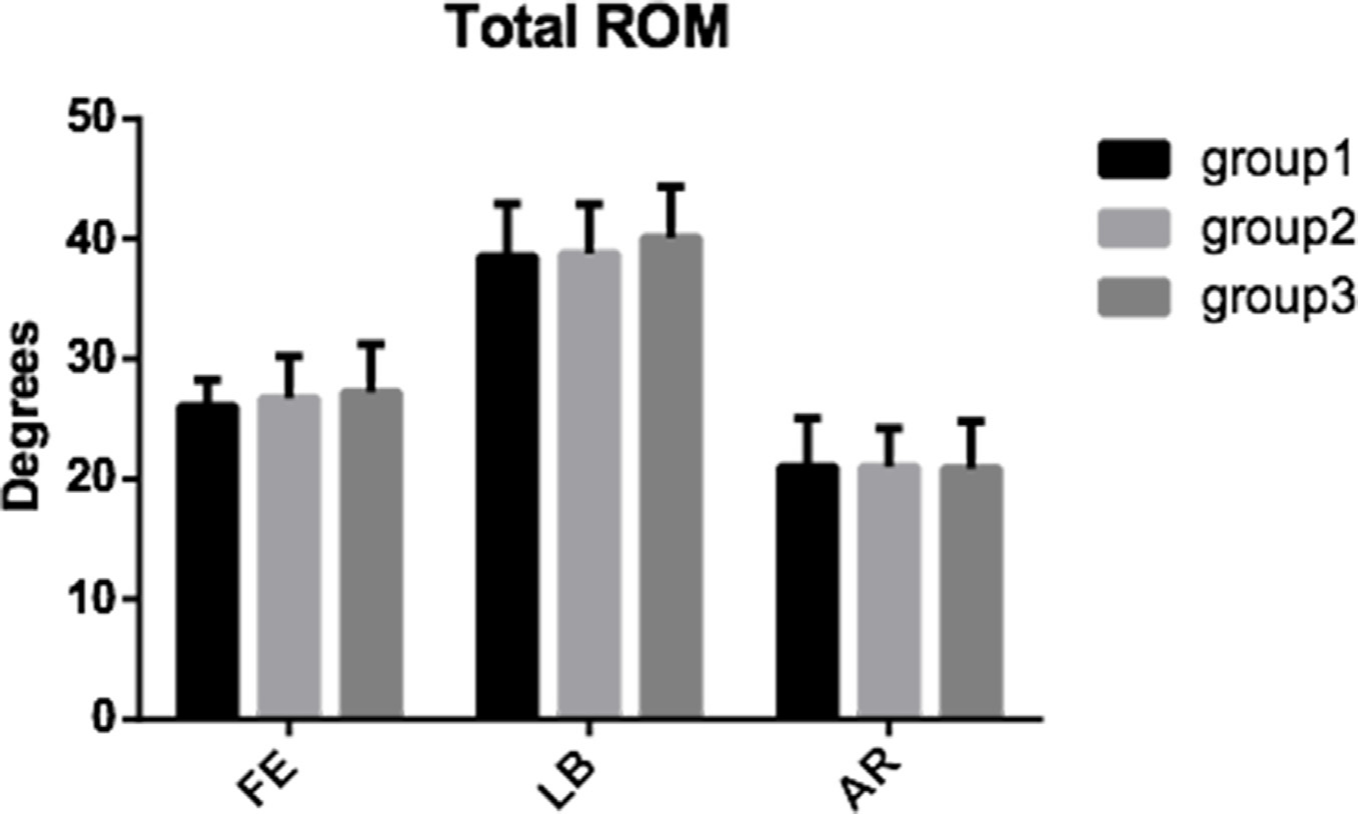
The total ROM. The total ROM of intact (group 1), C3/4 CDR with the novel prosthesis based on the physiological curvature of the endplate (group 2) and C3/4 CDR with the Prestige LP prosthesis (group 3) in all three motion directions.

The differences in total ROM among the three groups were not statistically significant in all three motion directions, and had the following values: for flexion-extension 26.07° ±2.18° (group 1), 26.72° ±3.51° (group 2) and 27.24° ±3.97° (group 3); for lateral bending 38.54° ±4.39° (group 1), 38.78° ±4.13° (group 2) and 40.10° ±4.25° (group 3); for axial rotation 21.02° ±4.04° (group 1), 21.04° ±3.17° (group 2) and 20.89° ±3.92° (group 3). The mean total ROM in three motion directions was always recorded at the maximum loading of ±2 Nm. As shown in Fig 5, the three groups had minor changes in the total ROM (p > 0.05).

**Fig 6:**
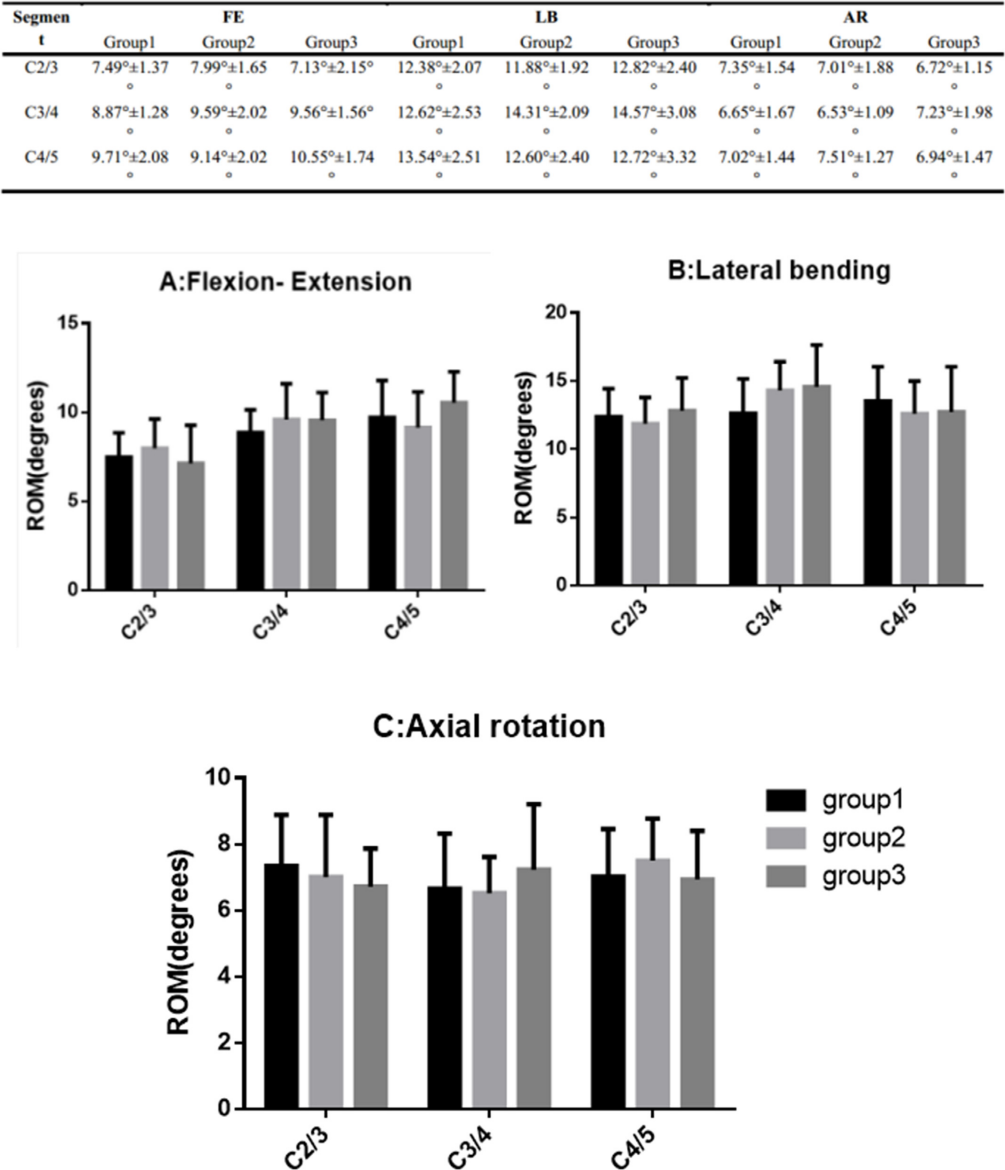
Segmental ROM. The ROM of the three groups in flexion-extension (A), lateral bending (B), and axial rotation (C).

The mean values of the ROM C2/3, C3/4 and C4/5 in all three motion directions of three groups are shown in Fig 6. The three groups had no significant differences in all segments in all three motion directions (p>0.05).

### Pressure analysis

**Fig 7:**
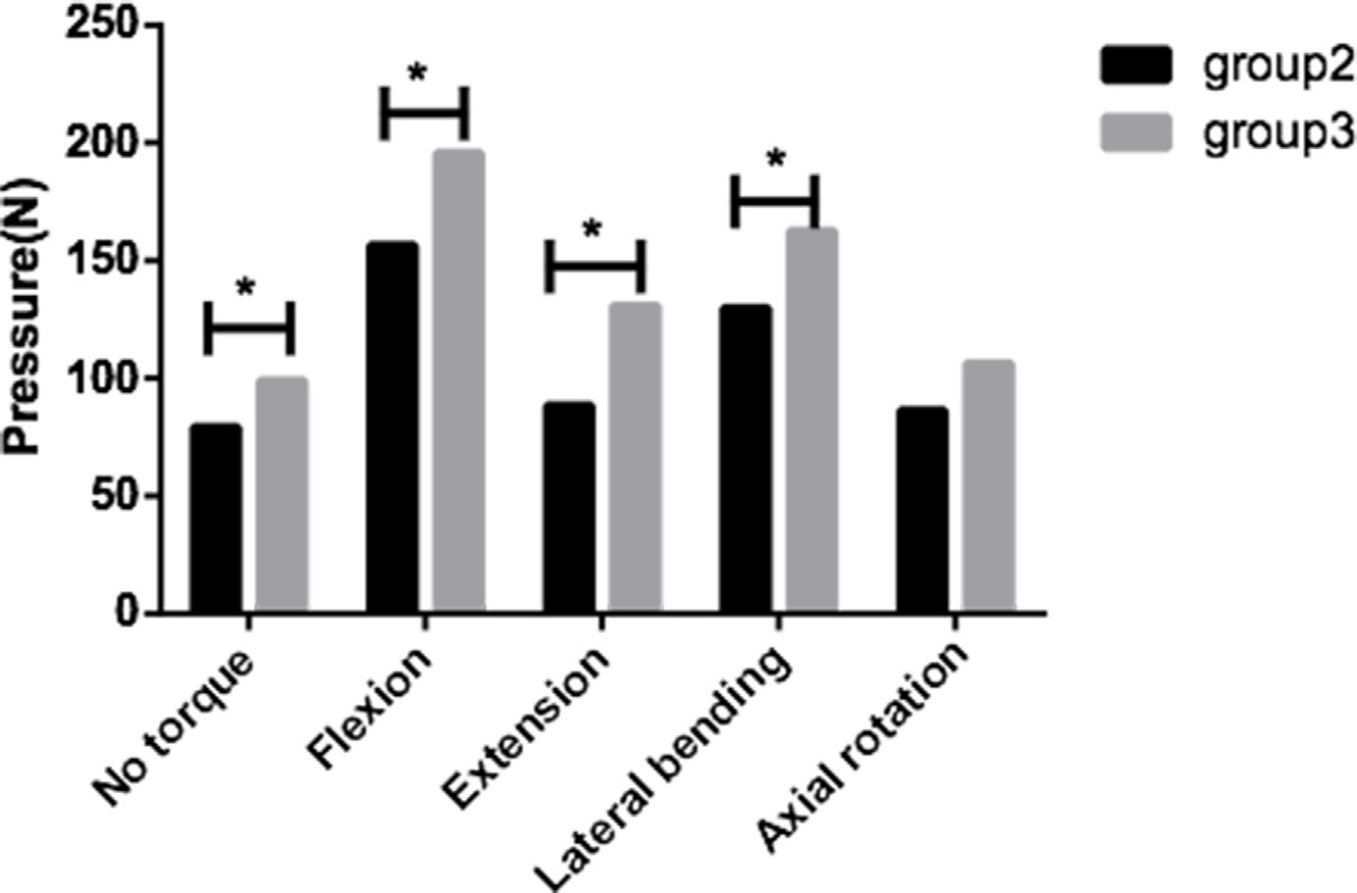
Maximum pressures. The maximum pressures on the inferior surfaces of the novel prosthesis and Prestige LP prosthesis. Statistically significant differences are denoted by *, with bars connecting the corresponding columns.

The pressures of the groups 2 and 3 were compared (Fig 7). The maximum pressures on the novel prosthesis’ inferior surface (79±18N) showed significantly (p<0.05) lower values at no torque, as compared to the Prestige LP prosthesis (99±18N). In flexion, the pressure was 157±27N in group 2, which was significantly lower than 196±36N of group 3 (p<0.05). In extension, group 2 (89±26N) also had a significantly lower pressure than group 3 (131±29N) (p<0.05). In lateral bending, the maximum pressures on the inferior surface of the two prostheses were 130±23N and 163±21N, respectively, which were significantly different from each other (p<0.05). The mean pressure in group 2 (86±19N) was slightly lower than in group 3 (106±19 N) in axial rotation (p>0.05).

## Discussion

Cervical fusion sacrifices segmental mobility and increases ROM stresses at the adjacent segment. Overloading at the adjacent segments caused by fusion contribute to adjacent segment degeneration [21–24]. Cervical disc replacement is a successful and promising non-fusion technique aimed at resuming normal articular motion and spine kinematics. The benefits of CDR over ACDF, the gold standard technique, have been demonstrated in several prospective randomized controlled trials [25–29].

As CDR is very promising in the operative management of degenerative disc disease in the cervical spine, *in vitro* studies are very important to analyze the biomechanics of the different implants. Human cadaveric specimens are very difficult to acquire for such studies and there are some disadvantages to human tissue testing, for instance the variability in age, gender, height, stiffness, bone quality, and grade of degenerative changes. Therefore, animal spines are commonly used to imitate the human spine. Although animal models (including sheep) differ both anatomically and biomechanically from the human spine, they provide a first step to assessing the benefits and drawbacks of surgical techniques and implant designs. A number of anatomic differences in the cervical spine were observed between the human and sheep: 1)human vertebral bodies were cylindrical, whereas sheep vertebral bodies had a conic shape; 2)human vertebrae were wider than tall, whereas sheep vertebrae were taller than wide; 3)and human pedicles were nearly round, whereas sheep pedicles were ellipsoid. Overall, these differences may affect the result of this study. Yet, fundamental similarities also existed between the human and sheep such as the bone mineral density of the middle and lower cervical spine. Tests on many animal spines have confirmed that the ROM of sheep is most similar to that of human [15]. Additionally, the ovine C2/C3 and C3/C4 segments are the most suitable for biomechanical testing.

From a perspective of biomechanics, an implant with the largest possible surface area appears best to avoid subsidence into the vertebral body, as the circumference would provide a brace for the strongest areas in the periphery. Inadequate endplate design can equally contribute to subsidence as a result of extremely concentrated stress [6, 30]. The novel artificial disc prosthesis has the arcuate surface which can get more effective contact area to the endplates than a flat surface. On each surface of the novel prosthesis are six denticulations and at the front of prosthesis are four baffles, which are all used to fix the prosthesis from migration. With the same loading derived from the superior vertebral body, the arcuate surface can bear less pressure than a flat surface just like the Prestige LP prosthesis. The reduced implant-bone interface pressure can decrease the risk of subsidence.

Previous studies had suggested that the application of a cervical follower load decreases motion and bending moments of functional spinal units. But we still used a follower load of 50 N, for increasing the clinical practicability. There are still controversies using displacement-controlled versus load-controlled protocol during the testing [31]. We thought the load-control testing mode may appear more physiologically representative as the weight of the head and muscle forces in the neck presumably do not change before and after surgery. Hence, we used load-controlled protocol for biomechanical tests in our study.

In this study, we tested the kinematics of cadaveric ovine cervical spines under three conditions (intact and CDR with two types of prostheses). Furthermore, the pressures on the inferior surface of two prostheses were analyzed. There were minor changes in the ROM after arthroplasty between the novel prosthesis and the Prestige LP prosthesis (p>0.05). Therefore, the novel prosthesis based on the physiological curvature of the endplate is similar to the Prestige LP prosthesis in simulating cervical disc. The mean pressure on the inferior surface of the novel prosthesis were significantly lower than the Prestige LP prosthesis in almost all situations, except axial rotation. The mean pressures of group 2 were slightly lower than group 3 in axial rotation, which may be statistically significant with a larger sample size. The pressure analysis test showed that the novel prosthesis can reduce the pressure on the inferior surface, and thereby decrease the incidence of subsidence. Similar to previous studies, the ROM in the segments after CDR in our study approximated the corresponding values under intact condition [32, 33]. In contrast, one study had shown significantly higher ROM in the adjacent segments after CDR [34].

As the limitation of any *in vitro* cadaveric biomechanical analysis of the cervical spine, our study focused mainly on the extent of motion without considering the quality of motion, and cannot represent the long-term effect of CDR. And the sheep model does not reflex real human spine. Additionally, no fatigue test was performed in this biomechanical analysis.

## Conclusion

ROM in three groups (intact group, CDR group with novel prosthesis and CDR group with Prestige LP) showed no significant difference. More importantly, prosthesis subsidence is one common complication after the CDR surgery, however the pressures on the inferior surface of the novel cervical artificial disc prosthesis were apparently lower than the Prestige LP prosthesis. Thus, it suggested that the novel artificial disc prosthesis could be exactly feasible and effective in CDR, could reduce the implant-bone interface pressure on the endplate, and particularly, lowered the risks of prosthesis subsidence.
